# Artificial Intelligence in Acute Kidney Injury Risk Prediction

**DOI:** 10.3390/jcm9030678

**Published:** 2020-03-03

**Authors:** Joana Gameiro, Tiago Branco, José António Lopes

**Affiliations:** 1 Division of Nephrology and Renal Transplantation, Department of Medicine, Centro Hospitalar Lisboa Norte, EPE, Av. Prof. Egas Moniz, 1649-035 Lisboa, Portugal; jalopes93@hotmail.com; 2 Department of Medicine, Centro Hospitalar Lisboa Norte, EPE, Av. Prof. Egas Moniz, 1649-035 Lisboa, Portugal; tiagobranco.md@gmail.com

**Keywords:** acute kidney injury, risk prediction, artificial intelligence

## Abstract

Acute kidney injury (AKI) is a frequent complication in hospitalized patients, which is associated with worse short and long-term outcomes. It is crucial to develop methods to identify patients at risk for AKI and to diagnose subclinical AKI in order to improve patient outcomes. The advances in clinical informatics and the increasing availability of electronic medical records have allowed for the development of artificial intelligence predictive models of risk estimation in AKI. In this review, we discussed the progress of AKI risk prediction from risk scores to electronic alerts to machine learning methods.

## 1. Introduction

Acute kidney injury (AKI) is a complex syndrome caused by multiple etiologies and characterized by a sudden decrease in kidney function, defined by an increase in serum creatinine or a decrease in urine output [1,2]. AKI is a frequent complication in hospitalized patients, which is associated with worse short and long-term outcomes, namely, increased length of hospital stay, increased health care costs, increased risk of in-hospital and long-term mortality, long-term progression to chronic kidney disease, and long-term risk of cardiovascular events [3,4,5,6,7].

The incidence of AKI has increased in the past decades due to the population aging and rising incidence of comorbidities, such as chronic kidney disease, diabetes, and hypertension [2,8,9,10]. Furthermore, the development of a standardized definition for AKI and the acknowledgment of the impact of AKI on patient outcomes are also responsible for the increased recognition of this syndrome [2]. Despite the decrease in mortality rates associated with AKI, these remain significant, ranging from 15% among hospitalized patients to more than 50% in critically ill patients [11,12,13].

Considering the impact of AKI on short and long-term outcomes, it is of high importance to develop methods to identify patients at risk for AKI and to diagnose subclinical AKI in order to improve patient outcomes [4]. The advances in clinical informatics and the increasing availability of electronic medical records (EMR) have allowed for the development of predictive models of risk estimation in AKI [14].

In this review, we discussed the progress of AKI risk prediction from risk scores to electronic alerts to machine learning (ML) methods.

## 2. AKI Definition and Biomarkers

The Kidney Disease Improving Global Outcomes workgroup defines AKI as an increase in serum creatinine (SCr) of at least 0.3 mg/dL within 48 h, or an increase in SCr to more than 1.5 times of baseline level, which is known or presumed to have occurred within the prior 7 days, or a urine output (UO) decrease to less than 0.5 mL/kg/h for 6 h [15].

Despite the importance of the development and use of this standardized classification in the epidemiology of AKI, SCr and UO are insensitive and unspecific markers of AKI, which do not account for the duration or cause of AKI [16]. Values of SCr are influenced by age, gender, muscle mass, fluid balance, and medications, which limit its secretion, and values of UO are influenced by patient volemic status and diuretic use. Baseline SCr is frequently unknown, and UO assessment is complex without a urinary catheter.

Novel biomarkers have been investigated in multiple settings to increase diagnostic accuracy, which so far include cystatin C (Cys-C), neutrophil gelatinase-associated lipocalin (NGAL), N-acetyl-glucosaminidase (NAG), kidney injury molecule 1 (KIM-1), interleukin-6 (IL-6), interleukin-8 (IL-8), interleukin 18 (IL-18), liver-type fatty acid-binding protein (L-FABP), calprotectin, urine angiotensinogen (AGT), urine microRNAs, insulin-like growth factor-binding protein 7 (IGFBP7), and tissue inhibitor of metalloproteinases-2 (TIMP-2) [17,18,19,20,21,22,23,24,25,26,27].

Important weaknesses have limited the generalization of the use of these biomarkers in clinical practice [19]. These have not consistently distinguished pre-renal from renal AKI; several patient characteristics and comorbidities can produce range variations that limit their validity; cost-effectiveness is limited due to the increased costs associated with these biomarkers and need for multiple assessments, and evidence of outcome improvement is still lacking [28,29]. Given the complexity of AKI, perhaps the use of a panel of several biomarkers covering different stages of the syndrome could provide a better understanding of its pathophysiology and identify future treatment targets [29,30].

## 3. AKI Risk Factors

Several investigators have focused on determining significant risk factors for AKI [31,32,33]. Both patient susceptibilities and exposures are important risk factors for AKI. Patient age is an important non-modifiable risk factor [34,35,36]. The loss of renal reserve and physiologic decline of glomerular filtration rate (GFR) may place older patients at risk for AKI [37,38].

Chronic kidney disease (CKD) is another major risk factor for AKI [38] The loss of autoregulation, abnormal vasodilation, susceptibility to antihypertensive agents and nephrotoxins, and the side effects of medication contribute to the development of AKI in CKD patients [38].

Patient comorbidities, such as diabetes mellitus, hypertension, cardiovascular disease, chronic liver disease, and chronic obstructive pulmonary disease, have also been identified as important AKI predictors [15,31,32,33,36,39,40]. It is also important to note that HIV infection is a risk factor, predisposing patients to AKI, given the increasing incidence of HIV-infected patients in the past decades. Furthermore, AKI remains an important predictor of mortality in these patients despite the decrease in the incidence of AKI with the widespread use of antiretroviral therapy [41,42].

Exposure to sepsis, surgery, nephrotoxins, and shock are specific modifiable factors, which contribute to AKI [8,15]. Large cohort studies focusing on critically ill patients report that the two most important causes of AKI are sepsis and surgery [9,10,43,44,45].

More recent research has reported that hyperuricemia, hypoalbuminemia, obesity, anemia, and hyperglycemia may be new predictors of AKI [36].

Uric acid can contribute to AKI in several settings due to intratubular crystal precipitation, but also by inducing renal vasoconstriction and impairing autoregulation, and due to proinflammatory and antiangiogenic effects [46,47,48,49].

Hypoalbuminemia has been used as a nonspecific marker of patient nutrition, inflammation, hepatic function, and catabolic state and has been reported as an independent predictor of AKI in multiple settings [50,51,52,53,54].

The increasing incidence of obesity has raised the interest to study its association with AKI. Although the exact mechanisms are still uncertain, there is substantial evidence that obesity is an independent predictor of AKI in multiple medical and surgical settings [55,56,57,58].

Anemia has been associated with increased AKI risk, mainly in the surgical setting [59,60]. Furthermore, transfusions of red blood cells are also associated with increased risk of AKI [61,62]. The mechanisms are likely multifactorial, including reduced renal oxygen delivery, worsening oxidative stress, systemic inflammation, and impaired hemostasis [36,59,63]. Stored red blood cells have an impaired ability to carry oxygen and proinflammatory effects, associated with the direct toxic effect of by-products of red blood cell storage, contributing to organ failure in critically ill patients [59,64].

Hyperglycemia is another novel risk factor, which has been associated with increased AKI development [65,66,67,68,69,70]. However, the target level of glucose to decrease AKI risk has not been determined. The exact mechanism is still uncertain, but hyperglycemia might contribute to AKI through stimulation of oxidative stress, vasoconstriction and reduced renal oxygen delivery, and volume depletion due to osmotic diuresis [68,70].

## 4. AKI Risk Scores

A precise risk prediction score should be able to identify at-risk patients and guide clinicians on performing further diagnostic tests and prompting preventive and/or treatment measures. A risk score is produced by the combination of independent predictors of AKI and assigning relative impact, ideally with external validation analysis [14].

Risk prediction scores for AKI have been reported in several clinical settings, mostly in critical care, surgery, and contrast-induced nephropathy [71,72,73,74,75,76,77]. Still, most AKI cases are reported in general hospital wards, and risk scores in this setting are scarce [78,79,80,81].

Most models include age, gender, baseline kidney function, comorbidities, such as chronic kidney disease, diabetes, liver failure, and heart failure, medication history, namely, diuretics, angiotensin-converting-enzyme inhibitors and angiotensin-receptor blockers, and intra-procedure data to predict the risk of AKI [76,82]. An ideal risk prediction score for AKI should include a combination of demographic, clinical, and biological factors, along with biomarkers [76,77].

Malhotra et al. developed an easily calculated risk prediction score for AKI in critical care patients [77]. This risk score combines chronic kidney disease, chronic liver disease, congestive heart failure, hypertension, atherosclerotic coronary vascular disease, acidemia, nephrotoxin exposure, sepsis, mechanical ventilation, and anemia and has demonstrated good calibration in the test and external validation cohorts [77].

Flechet et al. developed four prediction scores, which can be used successively, based on the clinical information available [83]. The variables included in the baseline risk score are age, baseline SCr, surgical or medical category, diabetes, and planned admission. For the admission risk score, it includes blood glucose, suspected sepsis, hemodynamic support, and previous risk score variables. On day 1, the risk score includes SCr, Acute Physiology And Chronic Health Evaluation (APACHE) II score, maximum lactate, bilirubin, hours of ICU stay, and previous risk score variables. The risk score to be used after the first day includes the previous risk score variables and the total amount of urine, urine slope, mean arterial pressure, and hemodynamic support. One of the main strong points of this study is the availability of the online calculator of this risk score, which enhances its use in clinical practice and promotes further validation [83].

The most widely validated risk prediction score for AKI in cardiac surgery was developed by Thakar et al. and comprises 13 pre-operative variables, namely, gender, heart failure, left ventricular ejection fraction, preoperative use of intra-aortic balloon pump, chronic obstructive pulmonary disease, diabetes, previous cardiac surgery, emergency setting, type of surgery, and pre-operative SCr [84].

The clinical application of these risk prediction scores has been limited by the lack of external validation of several studies, the use of heterogeneous definitions of AKI, the difficulty in assessing baseline renal function, and importantly the lack of impact analysis studies and lack of evidence of clinical use [14,76].

## 5. Automated Electronic Alerts

The use of automated electronic alerts (E-alerts) has received considerable consideration in the past years [85]. E-alerts consist of algorithms configured from patients’ EMRs and clinical information to notify early or imminent AKI, prompting an earlier clinical evaluation and prompt prevention and treatment strategies [86,87].

Theoretically, this would prompt early treatment and improve patient outcomes. Nevertheless, a recent systematic review of randomized AKI E-alert trials pooled data from six studies and 10,165 patients and found that these did not reduce mortality (OR 1.05; 95% CI, 0.84–1.31), need for renal replacement therapy (OR 1.20; 95% CI, 0.91–1.57), or change patient care practices (OR 2.18; 95% CI, 0.46–10.31) [88]. In these studies, E-alerts were issued within one hour, following the detection of changes in SCr; however, there was significant variability in study design, alert format, and targeted providers [88].

Beyond the limitations of SCr as a marker of AKI, other important challenges of the use of E-alerts are the distinction of community and hospital-acquired AKI cases, the presence of multiple alerts per patient, the assessment of significance of small SCr changes in patients with CKD, and the limitations on cases without baseline renal function [14].

A care bundle is a group of evidence-based and easily applicable interventions that have a better outcome when performed together than if performed individually [14]. There is no current specific treatment for AKI, and the most recent guidelines suggest supportive management, including treatment of sepsis, shock, and hypovolemia, avoidance of nephrotoxins, appropriate investigations, and referral to specialists when indicated [15].

Kolhe et al. demonstrated that implementing a care bundle with E-alerts improved outcomes in patients with AKI in two cohort studies. The care bundle consists of standardized investigations and interventions, namely, Assessment of history and examination, Urinalysis, establishing a clinical Diagnosis of AKI, plan Investigations, and Treatment and Seeking advice from a nephrologist (AUDITS) [89,90].

These findings were also reported in a study by Chandrasekar et al., in which an E-alert was combined with a care bundle consisting of treatment of Acute complications, Blood pressure control, Catheterization, review Drug prescription, Investigate the cause, and Treat the underlying cause (ABCD-IT) [91]. The authors reported a decrease in mortality and length of stay [91].

A recent study by Hodgson et al. evaluated the impact of combining care bundles to a risk prediction score and to E-alerts. This study demonstrated a decrease in hospital-acquired AKI and a decrease in AKI-associated mortality [92].

Therefore, it may not be enough to merely alert for the presence of AKI but important to initiate appropriate care to lead to improved outcomes [14].

We believe that it is essential to incorporate these scientific advances in daily clinical practice in the near future.

## 6. The Era of Artificial Intelligence

The past decade has seen significant development of electronic technology in medicine, namely, in EMR, data registries and management, and analytic methodologies [93].

Indeed, a new era of AKI prediction and detection has started with the increasing use of risk prediction scores and E-alerts [93]. More recently, artificial intelligence (AI), namely, ML techniques, has been reported to identify AKI predictors [94].

AI is a branch of engineering, generally defined as the ability of a machine to reason, communicate, and function with the minimal human intervention [95]. In the medical field, AI can be applied as two branches: physical or virtual [95]. The physical branch includes medical devices and sophisticated robots, which contribute to the delivery of care [95]. The virtual branch refers to ML, which includes the algorithms and statistical models that learn from data from which they are able to recognize and deduce patterns [95].

There are numerous types of ML algorithms, which have the ability to find patterns, to classify and predict algorithms based on previous examples, and to create a strategy for prediction by sequences of rewards and punishments [94,95,96,97,98]. The dynamic ability of these algorithms is key to identify and integrate variables from numerous electronic data [94]. Thus, ML techniques can be used alone or combined to analyze datasets and determine AKI predictors. The description of each available ML algorithm is out of the scope of this review.

Currently, logistic regression is the most frequently used statistical algorithm of multivariate analysis to determine risk predictors in the short-term [99]. In complex settings in which clinical features and outcomes have a non-linear relationship and for big data analysis, many investigators support the use of more advanced ML algorithms in detriment of logistic regression to develop predictive models [100].

Considering that AKI can be determined from the calculation of SCr levels and the increasing integration of the available EMRs, ML algorithms are promising in the development of AKI risk prediction models.

The development of risk prediction models has flourished in recent years. However, inefficient statistical methods, the use of small samples, missing data, and lack of validation are common faults, which limit the use of these models [99]. The development of risk prediction models should include internal validation within the original study sample to quantify the predictive ability of the model and should preferably also include external validation to evaluate the predictive ability of the model in other participant data [99]. To improve the quality of reporting of published prediction model studies, the Transparent Reporting of a multivariable prediction model for Individual Prognosis or Diagnosis (TRIPOD) Statement produced a checklist of items to include in studies developing or validating a multivariable prediction model [99].

Furthermore, it is important to consider the specificity and sensitivity of these models, which will have a clinical impact [101]. High specificity values lead to fewer false-positive results, and high sensitivity values lead to fewer false negatives [101]. This has an important impact on prognostic modeling and decision-making, namely, high specificity would trigger less often to prompt interventions with higher risk, and high sensitivity would trigger more often to prompt interventions with lower risk [101].

The first study to compare logistic regression models and ML algorithms was a retrospective study by Kate et al., who analyzed EMRs of 25,521 hospital stays of elderly patients and aimed to predict within the first 24 h of admission whether a patient would develop AKI during hospitalization. This study demonstrated only modest performance in all ML models (support vector machines, decision trees, and naïve Bayes), with an area under the receiver operating characteristic curve (AUROC) ranging from 0.621–0.664, and better performance of logistic regression with an AUROC of 0.743 [102].

A research group led by Bihorac performed a retrospective study of 50,318 adult surgical patients and compared four predictive ML modeling approaches for two major postoperative complications, using data from EMRs [103]. This study demonstrated that the choice of predictive modeling approach affected the risk prediction performance for postoperative AKI and sepsis; specifically, generalized additive models showed the best performance with an AUROC of 0.858 [103].

Davis et al. also compared several ML models (random forest, neural network, and naïve Bayes) and logistic regression methods to predict AKI in a retrospective study of 2003 patients [104]. Both methods had a good performance in detecting AKI, but importantly, over-time logistic regression methods required more updates than random forest or neural network methods to compensate overprediction [104].

Cheng et al. developed ML-based AKI prediction models using EMRs from 48,955 hospital admissions and concluded that the best model for predicting AKI within 24 h had an AUROC of 0.76 achieved by a random forest algorithm [105]. Indeed, this ML algorithm could predict AKI 2-days with AUC of 0.73 and 3-days prior with AUC of 0.700 [105].

Ibrahim et al. developed a clinical and proteomics AKI risk predictor with an ML approach (least absolute shrinkage and selection operator (LASSO) with logistic regression) in a prospective study of 889 patients undergoing coronary angiography [106]. The risk predictor included a history of diabetes, blood urea nitrogen/creatinine ratio, c-reactive protein, osteopontin, CD5 antigen-like, and Factor VII and had an AUROC of 0.790 for predicting procedural AKI [106].

Koola et al. analyzed a retrospective cohort of 504 cirrhotic patients and compared the ability of ML methods (logistic regression, naïve Bayes, support vector machines, random forest, and gradient boosting) to predict hepatorenal syndrome (HRS) [107]. This study demonstrated the ability to create a high-performance risk prediction algorithm to detect cases of HRS, with AUROC ranging between 0.730–0.930 [107].

Another mortality prediction model was constructed using the random forest algorithm in 19,044 AKI patients by Lin and colleagues [100]. Urine output, systolic blood pressure, age, serum bicarbonate, and heart rate were the most significant variables, predicting AKI-associated mortality [100]. This model had a great performance with an AUROC of 0.866 and could prove useful in avoiding delays of AKI treatment in high-mortality risk patients [100].

Koyner et al. developed a gradient boosting model, which could predict AKI in the emergency department, wards, and ICU [108]. Their model included data from 121,158 admissions, such as patient demographics, vital signs, laboratories, clinical interventions, and diagnostics, and demonstrated increasing accuracy across AKI severity, providing AUROC greater than 0.900 for renal replacement therapy requirement within 72 h [108].

Huang et al. performed a retrospective study of 947,091 patients submitted to percutaneous coronary intervention and compared logistic regression and gradient descent boosting to detect if ML algorithms could enhance AKI prediction [109]. Their algorithm had a good performance in detecting AKI with an AUROC 0.728 [109]. The risk prediction model included 12 variables, namely, age, heart failure, cardiogenic shock within 24 h, cardiac arrest within 24 h, diabetes, coronary artery disease, baseline renal function, admission source, body mass index, emergency status, and left ventricular ejection fraction [109].

In another retrospective study of 2,076,694 patients submitted to percutaneous coronary intervention, Huang et al. applied an ML method to predict AKI risk according to contrast volume [110]. The generalized additive model produced an AUROC of 0.777 (95% CI, 0.775–0.779) for predicting the risk of a creatinine level increase of at least 0.3 mg/dL [110]. The model was developed from a random 50% of the cohort, and performance was evaluated in the remaining 50% of the cohort. The association of contrast volume with AKI risk was nonlinear, and this model proved useful to quantify individual risk and adjust contrast volume to decrease AKI risk [110].

Tomasev et al. developed a recurrent neural network model, which predicted 55.8% of all inpatient episodes of AKI and 90.2% of all dialysis, requiring AKI up to 48 h in advance in 703,782 adult patients from inpatient and outpatient sites [111]. This ML model had a great performance with an AUROC of 0.921, and, at each time point, this model outputted the risk of AKI occurrence within the next 48 h, thus allowing for the prompt implementation of preventive and treatment strategies [111].

*MySurgeryRisk* is an ML algorithm recently developed and internally validated from a retrospective single-center cohort of 2911 adults who underwent surgery [112]. This random forest model combined preoperative and intraoperative variables and had an AUROC of 0.860 to predict the risk of developing postoperative AKI [112].

Flechet et al. conducted a prospective observational study of 252 critically ill patients and compared the AKI predictions by physicians and a random forest method, *AKIpredictor* [113]. There was no statistically significant difference in discrimination between physicians and *AKIpredictor;* however, physicians overestimated the risk, and *AKIpredictor* allowed for the selection of high-risk patients or reducing false positives, and *AKIpredictor* provided its prediction earlier than physicians [113].

Parreco et al. developed and compared different ML models (gradient boosted trees, logistic regression, and deep learning) to predict AKI from the laboratory values, vital signs, and slopes in 151,098 ICU admissions [114]. Gradient boosted trees method was the most accurate model with an AUROC of 0.834, for which the most important variable was the slope of the minimum creatinine [114].

Xu et al. investigated ML models (logistic regression, random forest. and gradient boosting decision tree) for predicting the mortality risk of 58,976 AKI patients admitted to an ICU, stratified according to AKI severity stages [115]. Gradient boosting decision tree presented a better performance than other models for mortality prediction [115].

Tran et al. developed an ML method (k-nearest neighbor) to predict AKI in 50 burn patients, which included measurements of neutrophil gelatinase-associated lipocalin (NGAL), UO, SCr, and N-terminal B-type natriuretic peptide (NT-proBNP) measured within the first 24 h of admission. This method performed greatly with an AUROC of 0.920 and achieved a 90%–100% accuracy for identifying AKI, with a mean time-to-AKI recognition within 18 h [116].

In 6682 critical care patients, Zhang et al. identified predictors of volume responsive AKI, such as age, urinary creatinine concentration, maximum blood urea nitrogen concentration, and albumin using ML methods [117]. Their model (gradient boosting) had an AUROC of 0.860 and could prove useful to stratify patients with oliguria responsive to fluids and prompt immediate therapeutic measures [117].

Zimmerman et al. conducted a retrospective cohort of 23,950 adult critical care patients and developed a predictive model by logistic regression for early prediction of AKI in the first 72 h. following ICU admission with an AUROC of 0.783 [118]. Their model included first-day measurements of physiologic variables but not medications and procedures, in order to detect which deterioration of patients’ physiologic baselines are predictive of AKI [118]. This was cross-validated with ML algorithms, demonstrating an accurate and early prediction of AKI with their risk prediction score [118].

Rashidi and colleagues developed, internally validated, and compared ML models for early recognition of AKI in 50 burn and 51 trauma patients, including NGAL, NT-proBNP, SCr, and UO into the predictive model [119]. Their models were able to accurately predict AKI 62 h in advance [119].

Overall, ML algorithms have performed impressively, and sensitivity is favored over specificity in order to early detect as many cases of AKI, allowing for a higher number of false positives. The ML algorithms have also performed better than the currently used logistic regression in the majority of studies. These studies are summarized in Table 1.

These studies have demonstrated the efficacy of ML algorithms to detect clinical and laboratory characteristics associated with AKI risk and detection in big data studies. The future widespread use of ML algorithms could improve risk stratification of patients, early detection of AKI, and provide decision aid on treatment, ultimately improving patient care and increasing time and cost-efficiency. Furthermore, these algorithms could predict further adverse events and long-term prognosis, therefore, providing useful information to establish an individualized follow-up plan.

Despite the promising results, important limitations have to be considered [82]. Firstly, most ML approaches have performed positively in retrospective cohorts, and prospective implementation of these methods is still challenging [95,101]. None of these studies have external validation, and the variability in the availability of EMRs across centers limits the widespread use of these models [95,101]. The development of these risk prediction models requires a substantial amount of data from EMRs and computer-assisted risk prediction [82]. Furthermore, to guarantee detailed information on comorbidities, physiological and laboratory parameters and medication, and electronic connections between community and hospital data are necessary [82]. Logistic regression models are more familiar to clinicians than ML models, limiting data interpretation [103]. It is also important to note that neural networks are developed and tested in the same dataset, which limits generalizability [95,101]. Additionally, from a legal and ethical perspective, the inability to clarify what contributes to decision-making in neural networks is an important restriction in these models, which is conflicting to general data protection requirements [95,101].

## 7. Conclusions

AKI has a significant negative impact on short and long-term outcomes; thus, it is crucial to develop methods to identify patients at risk for AKI and to diagnose subclinical AKI. The increasing amount of evidence is encouraging the real-time implementation of these ML risk models as this does not require additional AKI biomarker testing. Combining these risk prediction models with early care bundles in the future is likely to improve patient outcomes.

## Figures and Tables

**Table 1:** Machine learning studies on acute kidney injury (AKI) prediction.

Study	Design	Setting	N	AKI Definition	Timing of AKI	ML Algorithm	Predictive Ability	Sensitivity; Specificity; Confidence Interval	External Validation
Kate et al. (2016)	retrospective	medical and surgical	25,521	AKIN	during hospitalization	naïve Bayes; support vector machine; decision trees; logistic regression	AUROC 0.654 AUROC 0.621 AUROC 0.639 AUROC 0.660	75%; 61%; -	no
Thottakkara et al. (2016)	retrospective	surgical	50,318	KDIGO	post surgery	naïve Bayes; generalized additive model; logistic regression; support vector machine	AUROC 0.819 AUROC 0.858 AUROC 0.853 AUROC 0.857	77%; -; -	no
Davis et al. (2017)	retrospective	medical and surgical	2003	KDIGO	during hospitalization	random forest; neural network; naïve Bayes; logistic regression	AUROC 0.730 AUROC 0.720 AUROC 0.690 AUROC 0.780	-; -; 95% CI	no
Cheng et al. (2018)	retrospective	medical and surgical	60,534	KDIGO, AKIN, RIFLE	during hospitalization	random forest; AdaBoostM1; logistic regression	AUROC 0.765 AUROC 0.751 AUROC 0.763	69%; 71%; -	no
Ibrahim et al. (2018)	prospective	contrast	889	KDIGO	pre and post intervention	logistic regression	AUROC 0.790	77%; 75%: -	no
Koola et al. (2018)	retrospective	medical and surgical	504	KDIGO	during hospitalization	logistic regression; naïve Bayes; support vector machines; random forest; gradient boosting	AUROC 0.930 AUROC 0.730 AUROC 0.900 AUROC 0.910 AUROC 0.880	87%; 76%; -	no
Lin et al. (2018)	retrospective	ICU	19,044	KDIGO	during hospitalization	support vector machine	AUROC 0.860	-	no
Koyner et al. (2018)	retrospective	medical and surgical	121,158	KDIGO	24 h post admission	gradient boosting	AUROC 0.900	95% CI	no
Huang et al. (2018)	retrospective	PCI	947,091	AKIN	during hospitalization	gradient boost; logistic regression	AUROC 0.728 AUROC 0.717	-; -; 95% CI	no
Huang et al. (2019)	retrospective	PCI	2,076,694	AKIN	pre and post intervention	generalized additive model	AUROC 0.777	-; -; 95% CI	no
Tomašev et al. (2019)	retrospective	medical and surgical	703,782	KDIGO	during hospitalization	recurrent neural network	AUROC 0.921	95%; 70.3%; -	no
Adhikari et al. (2019)	retrospective	surgical	2901	KDIGO	post surgery	random forest	AUROC 0.860	68%; -; -	no
Flechet et al. (2019)	prospective	ICU	252	KDIGO	during hospitalization	random forest	AUROC 0.780	-; -; 95% CI	no
Parreco et al. (2019)	retrospective	medical and surgical	151,098	KDIGO	during hospitalization	gradient boosting; logistic regression; deep learning	AUROC 0.834 AUROC 0.827 AUROC 0.817	-; -; 95% CI	no
Xu et al. (2019)	retrospective	medical and surgical	58,976	KDIGO	during hospitalization	gradient boosting	AUROC 0.749	-	no
Tran et al. (2019)	prospective	burn	50	KDIGO	during hospitalization	k-nearest neighbor	AUROC 0.920	90%; -; -	no
Zhang et al. (2019)	retrospective	ICU	6682	KDIGO	24 h post admission	gradient boosting	AUROC 0.860	-; -; 95% CI	no
Zimmerman et al. (2019)	retrospective	ICU	46,000	KDIGO	72 h post admission	logistic regression; random forest; neural network	AUROC 0.783 AUROC 0.779 AUROC 0.796	68%; 34%; -	no
Rashidi et al. (2020)	retrospective and prospective	burn and trauma	50/51	KDIGO vs New Biomarkers	1st week post ICU admission	recurrent neural network	AUROC 0.920	-; -; 92% CI	no

AKI-acute kidney injury, AKIN-acute kidney injury network, AUROC-area under the receiver operating characteristic curve, ICU-intensive care unit, KDIGO-kidney disease improving global outcomes, ML-machine learning, PCI-percutaneous coronary intervention, RIFLE-risk, injury, failure, loss of kidney function, end-stage kidney disease.
